# Inferior vena cava resection and reconstruction with a peritoneal patch for a leiomyosarcoma: A case report

**DOI:** 10.1016/j.ijscr.2020.04.031

**Published:** 2020-05-08

**Authors:** Matteo Risaliti, Laura Fortuna, Ilenia Bartolini, Antonio Taddei, Paolo Muiesan

**Affiliations:** Department of Clinical and Experimental Medicine, University of Florence, AOU Careggi, Largo Brambilla 3, 50134, Florence, Italy

**Keywords:** CT, computed tomography, IVC, inferior vena cava, LMs, leiomyosarcoma, MRI, magnetic resonance imaging, US, ultrasound, ACS-NSQIP, American College of Surgeons - National Surgical Quality Improvement Program, FNCLCC, Fédération Nationale des Centres de Lutte Contre Le Cancer, PTFE, polytetrafluorethylene, Leiomyosarcoma, Inferior vena cava, Vascular reconstruction, Peritoneal patch

## Abstract

**Introduction:**

Leiomyosarcomas (LMs) of the inferior vena cava (IVC) are very rare neoplasms seldom reported in the literature. The majority of patients does not present with specific abdominal pain and IVC LMs are used to become symptomatic with the increase of tumor volume. The role of chemotherapy or radiotherapy is not yet defined and surgical resection seems to be the only chance to improve survival rates.

**Presentation of case:**

We present a case of a 58-year-old female with a recent diagnosis of IVC LM who underwent surgery with a partial resection of the anterior wall of the vein using a lateral and partial vein clamping. The primary repair of the defect could result in stricture of the vein, so a parietal peritoneum patch was used for the vein reconstruction. The postoperative course was uneventful.

**Discussion:**

Actual evidence suggests that vascular sarcomas have limited responsiveness to cytotoxic chemotherapy and chemoradiotherapy, so surgery is the treatment of choice. Major surgery entailing multivisceral and complex vascular resection is usually necessary to achieve negative margins and accurate vascular reconstruction techniques are mandatory to avoid serious circulatory complications. Different kinds of graft (biological or synthetics) are available for the reconstruction, with intrinsic advantages and limitations. The use of peritoneal patches seems a valid and cheap option for vascular reconstruction and it is gaining great attention in recent years.

**Conclusion:**

This case demonstrates that peritoneal graft could be a safe option to manage IVC defects in expert hands. A brief review of literature is also included.

## Highlights

• Leiomyosarcoma of the inferior vena cava are very rare neoplasm with a limited responsiveness to medical therapy.
• Surgery seems to be the only chance of cure.
• Complex vascular resection are often necessary and required accurate reconstruction technique.
• Peritoneal patch is an efficient and safe option to repair venous defects and it is gaining great attention in recent years.

## 1 Introduction

Vascular leiomyosarcomas are rare tumors and account for about 1–2% of all the sarcomas of soft tissue [1]. LMs arising from the IVC are even rarer and they are the cause of less than 1 case of malignancy in the adults every 100,000 [1]. However, their incidence may be underestimated since more than 50% of cases are diagnosed at autopsy [2]. Women are affected in 75–80% of the cases and LMs are predominant in the fifth-sixth decade of life at the time of diagnosis.

The role of chemotherapy or radiotherapy is not yet defined and surgical resection seems to be the only chance to improve survival rates.

Major surgery entailing multivisceral and complex vascular resection is usually necessary to achieve negative margins and accurate vascular reconstruction techniques are mandatory to avoid serious circulatory complications. Different kinds of graft (biological or synthetics) are available for the reconstruction, with intrinsic advantages and limitations. The use of peritoneal patches seems a valid and cheap option for vascular reconstruction and it is gaining great attention in recent years.

The current case report follows the Surgical Case Report (SCARE) guidelines [3].

## 2 Presentation of case

**Fig. 1:**
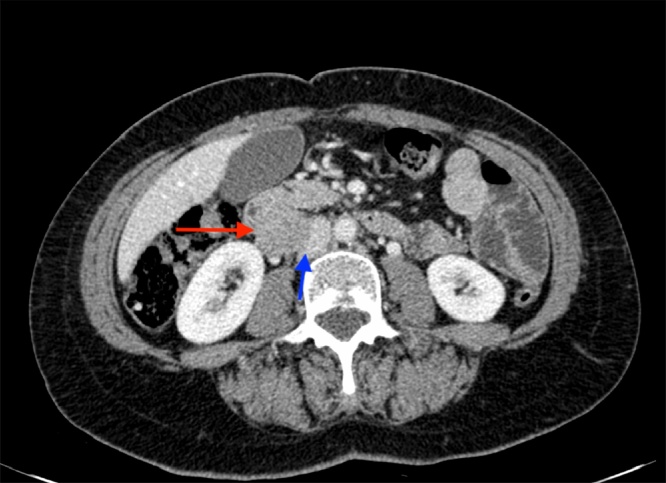
A contrast enhanced abdominal CT scan image. Red arrow pointing to the leiomyosarcoma and blue arrow pointing to the compressed IVC (For interpretation of the references to colour in this figure legend, the reader is referred to the web version of this article).

A 58-year-old female with a recent history of chronic abdominal pain was referred to our tertiary care center. An abdominal CT scan showed a mass of 3.5 cm between the inferior vena cava and the duodenum (Fig. 1). Chest CT scan was normal. Endoscopic ultrasound with biopsy was performed for further characterisation. Histopathology showed a spindle-cell malignant neoplasm with smooth muscle differentiation, consistent with a leiomyosarcoma.

After multidisciplinary evaluation, the patient was offered surgery. A midline incision was performed. After a medial rotation of the right-sided viscera (Cattell-Braasch maneuver) and a Kocher maneuver, we exposed the infrahepatic IVC and renal veins.

Intraoperative exploration revealed that the mass originated from the anterior aspect of the lower segment of the IVC with no infiltration of adjacent structures except for the right gonadal vein.

**Fig. 2:**
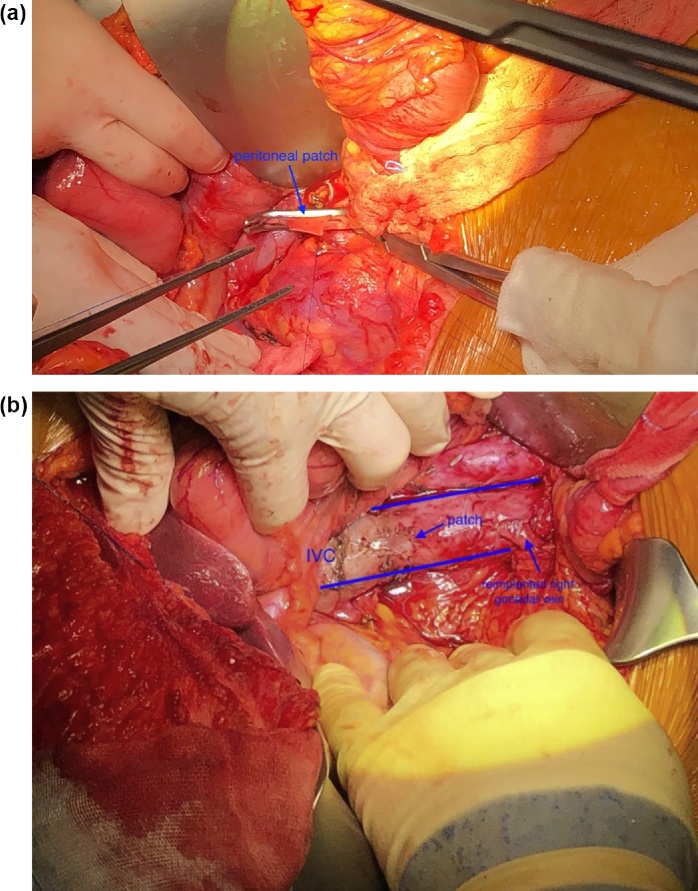
**a**. Intraoperative image. A lateral clamp on the IVC allows the blood flow to continue through the vein. After the removal of the mass with a portion of the surrounding vein, the peritoneal patch is hand-sewn to the vein. **b**. Intraoperative image. Final results after declamping the vein. The graft covered perfectly the vascular resection and there is not stricture of the vein. Right gonadal vein has been reimplanted.

A lateral clamping of the IVC was applied and the tumor was resected. The right gonadal vein was intimately attached to the tumor and, consequently, it was divided. Caval defect was about 1 cm and the direct suture was considered at risk of stenosis. For caval reconstruction, a rectangular peritoneal patch with the same dimension of the defect was harvested from the right parietal peritoneum. With gentle manipulation, all the fat tissue was removed from the graft and the patch was sutured with two 5/0 prolene running sutures with the mesothelial aspect applied to the lumen of the vessel (Fig. 2a). A heparinized solution was injected into the vessel and vascular clamp was then removed with good results. The distal stump of the right gonadal vein was reimplanted (Fig. 2b).

Postoperative course was uneventful and the patient was discharged in fifth postoperative day. No symptoms or complications were observed on the postoperative follow up.

Surgical pathological examination demonstrated a grade III leiomyosarcoma of the inferior vena cava of 5 cm of maximum diameter according to the FNCLCC (Fédération Nationale des Centres de Lutte Contre Le Cancer) system with a focally positive margin.

No evidence of disease at the time of this report, 5 months after surgery.

## 3 Discussion

Inferior vena cava LMs are seldom reported in literature and most of the papers are case reports or case series [4]. The scientific article with the widest number of patients is a pooled data analysis of previously published reports including 377 cases of IVC LMs [5]. Due to the rarity of this disease, treatment of choice is still debated. However, the actual evidence suggests that vascular sarcomas have limited responsiveness to cytotoxic chemotherapy and chemoradiotherapy [6] and, consequently, surgery is the treatment of choice when technically feasible for fit patients in absence of distant metastasis.

Without surgical resection, the prognosis is poor with an overall survival of months [1,5,6].

On the contrary, a chance of a 5-years overall survival rate of about 55% has been reported when a R0 resection is performed. However, recurrence rate is very high and 5-years disease-free survival rate is reported to be less than 10%.

En-bloc resection of adjacent organs may be necessary to achieve tumor-free margins. Despite the effect of en-bloc resection on long-term survival has not well defined, an analysis of the ACS-NSQIP (American College of Surgeons - National Surgical Quality Improvement Program) database reported that multi-organ resection for retroperitoneal sarcoma did not increase 30-day morbidity or mortality [7]. Furthermore, Watchel et al. in their article support the role of en-bloc resection in appropriate cases because the risk of positive margins affects the overall survival more than twice the surgical risk associated with an en-bloc resection [5].

If surgery is performed, the IVC can be safely ligated only in rare cases of slow-growing sarcomas causing complete obstruction when collateral veins could develop and bypass IVC block. However, low extremity oedema is a frequent complication [8,9].

In most of the cases, IVC control can be achieved with a lateral incomplete caval clamping with modest interruption of vascular return.

In case a complex and time-consuming vascular reconstruction is required, a venovenous bypass can be used to guarantee venous return to the heart particularly in older patient with compromised cardiac function [5].

Only few cases in literature report the use of cardiopulmonary bypass with full circulatory arrest: the limited use of this procedure reflects the fact that IVC sarcoma extending to the right atrium are likely to be considered borderline resectable because of locally advanced disease.

To avoid strictures of the vein, IVC primary repair without graft interposition is safe only when a small part of the vessel is involved by the tumor. A narrowing of less than 20% is considered acceptable [4].

In case of larger defects a replacement graft is recommended. Graft reconstruction has demonstrated excellent patency rates (80–90% after 5 years) with minimal complications [10].

Synthetics materials such as Dacron® or polytetrafluorethylene (PTFE) are safe and have been used with good results but both have inherent drawbacks such as the risk of thrombosis or infections requiring long term anticoagulation, antibiotic therapy and, sometimes, the need of graft removal [11].

Allografts such as cadaveric vein may be the most appropriate graft in term of low rates of complications, but their use is limited by their unavailability and high costs [12,13].

Autologous grafts such as saphenous or jugular vein grafts require a preoperative planning with further evaluation such as an ecocolordoppler; moreover, additional incisions are required [14].

Peritoneal patches are an efficient and safe option to repair venous defects and their use has been recently reported [12,15]. They can be easily and rapidly retrieved even in case of emergency situations, and additional preoperative planning or other skin incisions are not required.

Moreover, peritoneal patches are free and they can be used for temporary repair in selected cases when a better graft is available and being prepared.

Thickness of the patch could be different based on to the site of harvesting to best fit the vessel needing a repair: thinner patches may be harvested from the falciform ligament while thicker patches may be retrieved from the lateral hypochondrium with or without a muscular or aponeurotic layer.

Both allo- and autograft have demonstrated fewer thrombotic complications when compared to synthetic patches [16].

In particular, a recent study analyzed the risk of thrombosis in peritoneal patch compared to other autologous graft: vein intima may be injured due to repermeabilization-dilatation increasing the risk of thrombosis whereas mesothelium is rarely injured during the harvesting with a lower incidence of thrombosis [12,14].

On the contrary, peritoneal patches are less useful in case of larger or circumferential defects in which a synthetic or cadaveric graft could be more appropriate.

## 4 Conclusion

In conclusion, IVC LMs are rare retroperitoneal neoplasm with a poor prognosis and surgical resection is the only chance to improve patient survival. Vein reconstruction is often required. Different materials have been used, both synthetic or homologous.

Homologous grafts have less infectious and thrombotic complications and they can be at surgeon’s disposal also in emergency situations.

Peritoneal patches have been recently reported as a valid option to repair IVC defects in expert hands. In particular, they are easily and rapidly available and their use seems safe.

## Declaration of Competing Interest

The authors declare that there is no conflict of interest.

## Funding

This report received no specific grant from any funding agency in the public, commercial, or not-for-profit sectors.

## Ethical approval

Ethical approval was not required and patient-identifying knowledge was not presented in the report.

## Consent

Written informed consent has been obtained from the patient for the publication of this case report and accompanying images. A copy of the written consent is available for review by the Editor-in-Chief of this journal on request.

## Author contribution

Matteo Risaliti and Laura Fortuna: Conceptualization, Writing - original draft.

Ilenia Bartolini: Writing - Review, Editing and Supervision.

Paolo Muiesan and Antonio Taddei: Review, Editing, Supervision, Performed surgery.

## Registration of research studies

Not applicable.

## Guarantor

All authors were involved in the preparation of this case report.

## Provenance and peer review

Not commissioned, externally peer reviewed.
